# Impact-Aware Foot Motion Reconstruction and Ramp/Stair Detection Using One Foot-Mounted Inertial Measurement Unit

**DOI:** 10.3390/s24051480

**Published:** 2024-02-24

**Authors:** Yisen Wang, Katherine H. Fehr, Peter G. Adamczyk

**Affiliations:** Department of Mechanical Engineering, University of Wisconsin–Madison, Madison, WI 53706, USA; ywang2557@wisc.edu (Y.W.); kfehr@wisc.edu (K.H.F.)

**Keywords:** integration reconstruction, inertial sensors, ramp/stair detection, heel strike

## Abstract

Motion reconstruction using wearable sensors enables broad opportunities for gait analysis outside laboratory environments. Inertial Measurement Unit (IMU)-based foot trajectory reconstruction is an essential component of estimating the foot motion and user position required for any related biomechanics metrics. However, limitations remain in the reconstruction quality due to well-known sensor noise and drift issues, and in some cases, limited sensor bandwidth and range. In this work, to reduce drift in the height direction and handle the impulsive velocity error at heel strike, we enhanced the integration reconstruction with a novel kinematic model that partitions integration velocity errors into estimates of acceleration bias and heel strike vertical velocity error. Using this model, we achieve reduced height drift in reconstruction and simultaneously accomplish reliable terrain determination among level ground, ramps, and stairs. The reconstruction performance of the proposed method is compared against the widely used Error State Kalman Filter-based Pedestrian Dead Reckoning and integration-based foot-IMU motion reconstruction method with 15 trials from six subjects, including one prosthesis user. The mean height errors per stride are 0.03±0.08 cm on level ground, 0.95±0.37 cm on ramps, and 1.27±1.22 cm on stairs. The proposed method can determine the terrain types accurately by thresholding on the model output and demonstrates great reconstruction improvement in level-ground walking and moderate improvement on ramps and stairs.

## 1. Introduction

Wearable sensors are becoming increasingly reliable and convenient in terms of size, accuracy, battery life, robustness, range, data rate, and other performance measures. This development facilitates the deployment of wearable sensors in real-world studies where traditional motion capture systems cannot be used. As an attractive application, collecting foot kinematic data outside laboratory environments in different terrains, including ramps and stairs, can offer valuable data to enable scientific findings in gait analysis, such as understanding human locomotion [1,2,3,4,5], monitoring gait impairment [6,7], and in rehabilitation device control [8,9]. 

To reconstruct the foot trajectory, the Pedestrian Dead Reckoning (PDR) algorithm is often used to estimate the foot position and orientation from a foot-mounted Inertial Measurement Unit (IMU). It estimates the system states from linear and angular motion of the IMU and bounds the error accumulated between strides by applying Zero Velocity Updates (ZUPT) at the stance phase. PDR algorithms can be generally categorized into two types: integration methods [1,2,3,10,11,12,13,14] and filter-based methods [15,16,17,18,19,20,21]. The PDR algorithm can be further integrated with other sources of data like Global Navigation Satellite System (GNSS) [22,23], cameras [24,25], and other sources of data to further improve accuracy. 

A well-known issue in IMU-based PDR is that the position estimation suffers from drift in the vertical direction [11,19,26] in general and drift in the heading direction [19,27] in the absence of global information such as GNSS or reliable magnetometer data. The required accuracy of heading and vertical drift varies depending on specific applications. For example, in emergency rescue navigation [11], both heading and height error matter in identifying the location and floor of a building under restricted environments; in gait analysis using wearable IMU sensors, height drift matters if we want to evaluate foot clearance [13,14]. Noise and bias in IMU signals are usually considered as the main causes of drift [24,28,29], but limited sensor bandwidth or range might be another source of error [1,18,27,30]. Specifically, limited IMU sensor bandwidth cannot capture all the information in acceleration and angular velocity measurement under highly dynamic conditions [18,27]. A bandwidth above 100 Hz for acceleration and a bandwidth above 50 Hz for angular velocity are favored [27], while in reality, most consumer-grade Micro-Electromechanical Systems (MEMS) IMUs for out-of-lab data collection run at around 100 Hz. For example, the APDM Opal used in this study runs at 128 Hz, which means the bandwidth is only 64 Hz or less. Moreover, mathematically, the Kalman Filter is only derived to be optimal under white Gaussian noise [31,32], while in reality, the noise might not be Gaussian and therefore might degrade the filter performance [32,33]. A notable case is the impact error during heel strike. The impact signal is typically a non-Gaussian impulsive signal that will deteriorate the performance of the Kalman Filter [34]. In addition, the heel strike impact is often too short for the IMU to record [18]. As shown by [35] in the case of running, the impact signal can cause inaccurate velocity measurement, with severity depending on how much information is lost. During normal walking, heel strike impact may still be non-negligible depending on the pattern of walking and the characteristic of IMU sensor. One goal of this work is to mitigate the accumulated error in the vertical velocity and position due to unmodeled noise and missing information during heel strike impact. 

In some Inertial Navigation Systems (INS), the stochastic IMU error is modeled as a white noise term plus a bias term [28,29,36,37,38,39]. The second term is sometimes referred to as bias instability and modeled as a first-order Gauss–Markov process [29,37,38,39]. The Kalman Filter can model this bias term by adding extra filter states in its process model; these added states are called accelerometer bias and gyroscope bias, as shown in [28,36,39]. Building on this error model, and with the hope to estimate the heel strike error mentioned above without a Kalman Filter, the work presented here harnesses the intuition that we can separate the effect of acceleration bias on each direction and model the heel strike error explicitly as a vertical velocity impulse error, and thereby enhance the performance of widely used integration-based reconstruction [1,2,3,11,12,13,14] by reducing the drift in height. 

In this paper, we propose a kinematic model to estimate the acceleration bias and heel strike vertical velocity error, introduce an enhanced integration method to overcome this error in reconstructing the foot trajectory, and simultaneously detect ramp, stair, and level-ground walking, all using only one foot-mounted IMU. 

The contributions of this paper are the following:

(1) Propose a model to estimate acceleration bias and heel strike velocity error.
(2) Propose a novel method to detect ramp and stair locomotion (vs. level walking) using only one foot-mounted IMU.
(3) Propose an enhanced integration reconstruction method with improved accuracy in the vertical direction.

The structure of this paper is organized as follows: Section 2 states the related work on detecting ramps and stairs using wearable sensors. Section 3 introduces the kinematic model for estimating acceleration bias and heel strike velocity error. Section 4 describes the methods for detecting ramps and stairs. Section 5 states the enhanced integration reconstruction algorithm. Section 6 shows our human subjects experiment results. Finally, Section 7 presents the discussion and future work.

## 2. Related Work

Integration-based PDR using foot-mounted IMUs has proven very successful for tracking location in horizontal planar movement (e.g., x/y or latitude/longitude directions). For example in [1,2], the orientation of the IMU is first estimated by a Kalman Filter, then used to convert the local IMU acceleration into global acceleration, which is then double-integrated to obtain position. The orientation can also be estimated by pure strap-down integration of gyroscope data [11,12], with inclination correction from accelerometer data [3,10,40]. To bound the drift, the ZUPT is applied on velocity during each stance phase. However, foot-mounted IMUs are known to suffer from drift in the vertical direction when using the traditional ZUPT-based reconstruction. Compared to horizontal displacement errors on the order of 0.5–1% of stride length [11,18,26,41], higher vertical errors around 1–2% are common [11,18,19,21,26]. Vertical error is especially important in interpreting the reconstructed movement because of the influence of ground incline on biomechanical outcomes such as limb and joint mechanics [42,43,44,45,46], muscle behavior [47,48], and energetic cost [49]. Moreover, the direction can be either rising or falling in different circumstances but tends to be consistent within specific bouts and behaviors. This vertical drift is attributed to the vertical-direction impact at initial foot contact, which varies in magnitude and frequency content with speed and other movement characteristics. When locomotion data are known to take place on level ground, height de-drifting has been used to achieve reasonable reconstruction based on the assumption that floor height never changes [1,3,10], though this approach does not match the nature of the error and can cause the unrealistic changes in the reconstructed height movement. Similarly, if the slope of a ramp or dimensions of a staircase are known or can be identified in a database, appropriate de-drifting can also be applied [2,50]. But, in unconstrained, unsupervised real-world locomotion, it is impossible to know a priori whether certain strides are on level ground, ramps, stairs, or other terrain. In these cases, distinguishing ramps, stairs, and level walking is necessary to separate large data sets into different behaviors; however, this task can prove to be very difficult—particularly differentiating ramps vs. level walking—due to the uncertainty in vertical movement estimation. 

In general, activity recognition is a wider topic than PDR. Based on the sensor information used for the classification task, it can be categorized into dynamic-information-based [51] and trajectory-geometry-based activity recognition. Dynamic information includes ground reaction force, pressure, acceleration, and velocity. Typical preprocessing methods such as filtering and detecting gait cycles are applied, then features are extracted either in the time domain, such as sliding mean and sliding variance [52], peak magnitude, or histograms [53], or in the frequency domain, such as spectrograms [54]. However, those dynamic signals are correlated with walking speed [51] and may need assumptions like steady walking state.

On the other hand, trajectory-based information, which refers to the actual foot trajectory from IMU reconstruction or motion capture or the joint angle movement trajectory during each gait cycle, are less sensitive to walking speed. Bartlett and Goldfarb [9] classified activities by defining a phase variable from the thigh angle using a single IMU on the leg and matching the phase trajectory to the most similar averaged activity trajectory. The foot-mounted IMU trajectory in the sagittal plane can also be used to classify locomotion mode into level, stairs, and ramps by evaluating the inclination grade after some displacement from the prior footfall location, e.g., when the foot trajectory crosses an elliptical boundary [51]. One potential drawback is that imperfect reconstructions lead to overlap among trajectories from different activities [51]. Improvements to the accuracy of height trajectory reconstruction would be valuable for improving these methods, with impact on gait analysis, detection and quantification of impaired conditions, and control of robotic prostheses and exoskeletons.

## 3. Kinematic Model

As a core component of PDR algorithms based on IMU data and Kalman Filter architecture, the IMU error model decides the way of designing the filter states and characterizing process noise. We adopted the widely used IMU error model shown below as in [28,36].

(1) $$a_{t}=R\left(a_{m}-a_{b}-a_{n}\right)+g_{t}$$

(2) $$\omega_{t}=\omega_{m}-\omega_{b}-\omega_{n}$$

Here, the true IMU accelerometer and gyroscope measurement $a_{t},\omega_{t}$ is the combination of measured values $a_{m}(k),\omega_{m}(k)$ at each sample k, plus white noise term $a_{n},\;\omega_{n}$ and slow-varying accelerometer bias term $a_{b},\omega_{b}$. R is the rotation matrix for the IMU’s orientation in the world reference frame, and $g_{t}$ is the gravitational acceleration in the world frame.

The kinematic equations which model the linear and angular motion of the IMU sensor are shown as follows.

(3) $$p(k)=p(k-1)+v(k-1)∆t+\frac{1}{2}R(k)\left(a_{m}(k)-a_{b}\right){∆t}^{2}$$

(4) $$v(k)=v(k-1)+\left(R(k)\left(a_{m}(k)-a_{b}\right)+g\right)∆t$$

(5) $$q(k)=q(k-1)*q\left\{\left(\omega_{m}(k)-\omega_{b}\right)∆t\right\}$$

where the “*” operator stands for the quaternion multiplication, and $q\left\{∆\theta \right\}$ stands for the quaternion $\left[\begin{array}{cc} cos(\left|\frac{∆\theta }{2}\right|) & \frac{∆\theta }{\left|∆\theta \right|}sin(\left|\frac{∆\theta }{2}\right|) \end{array}\right]$ mapping from rotation vector ∆θ, represented as $\left(\omega_{m}(k)-\omega_{b}\right)∆t$. Vectors $p,v\in R^{3}$ are the position and velocity, and q is the quaternion associated with the orientation of the IMU, both represented in the world frame.

Because of the noise and bias terms in the IMU signal, at the end of each stride, the computed residual velocity will not be exactly zero. To handle this issue, the Zero Velocity Update (ZUPT) is used for correcting nonzero residual velocity. The basic idea is to assume the foot has zero velocity during the foot-flat phase and use this assumption to correct the drift in the velocity estimation. In integration-based reconstruction, the raw IMU data is first segmented to different strides, then the PDR states are integrated through each stride according to Equations (3)–(5). At the foot-flat phase in between each stride, the ZUPT is used to correct the drift: the residual velocity is modeled as a parameterized growing term with time since the prior ZUPT and subtracted from the computed velocity to obtain a zero end velocity [1,3,11,12,13,14]. In Kalman Filter-based reconstruction, the drift correction is implemented as a zero velocity pseudo-measurement update during the foot-flat phase for the filter, with the correction propagating to position, velocity, and other states based on the Kalman gain [15,28,50]. Multiple variants of Kalman Filter exist, including Extended Kalman Filter (EKF), Error State Kalman Filter (ESKF), and Unscented Kalman Filter (UKF). In the remainder of this paper, ESKF-based PDR [4] is selected for comparison against the proposed method. Instead of linearizing the nonlinear system dynamics like EKF and being affected by the linearization error [31], ESKF acts on the small error states of the system that have linear error propagation dynamics, thus gaining improved accuracy and robustness [28,31,39]. ESKF maintains a simple estimation of the nominal states and periodically injects error states into nominal states when filter measurements update is available, such as ZUPT. 

ZUPT is a key component in the PDR as it allows the algorithm to correct the error in between each stride, thus reducing the drift in the position from cubic in time into growth of lower order [55]. However, the linear correction still leaves error on the positions and the height tends to drift along the vertical direction [11,18,21], thus hindering the acquisition of accurate height information and, consequently, ground slope and stair kinematics and detailed foot–ground kinematics such as ground clearance estimates. For known level ground, a zero height change assumption can also be used to de-drift the height error over each stride [1,3,10,12], but then some of the key problems include (i) how to accurately detect the ramps and stairs with a minimal set of sensors, and (ii) how to re-implement height reconstruction for stairs and ramps, which are not compatible with the added assumption. The proposed method addresses these two problems at the same time. 

### 3.1. Linear and Impulsive Zero-Velocity Correction

The static bias in the gyroscope signal $\omega_{b}$, which is the main component of gyroscope bias with the biggest effect on orientation estimation [56], can be easily removed through estimation during any long stationary period. The accelerometer bias is harder to estimate from static calibration. In practice, the accelerometer bias often causes a significantly larger error compared with gyroscope bias, so we keep only the accelerometer bias term in the error state. We also switch the name and notation of accelerometer bias $a_{b}$ to acceleration bias ${b=\left[b_{x},b_{y},b_{z}\right]}^{T}$ to reflect a slightly different meaning—it covers more than just accelerometer bias in the IMU error model. It also accounts for noise, errors induced by walking pattern, and imperfections in orientation estimation (see further discussion in Section 7). The missing information in the heel strike event is modeled as a vertical velocity impulse, which we call *heel strike (HS) vertical velocity error*. In the following section, a kinematic model to estimate the acceleration bias and HS vertical velocity error is proposed. Note that the acceleration bias is defined in the IMU local frame while the HS vertical velocity error is defined vertically in the world frame.

Suppose the IMU orientation in each moment is already known (e.g., from any orientation estimation algorithm), and it has been used to transform the measured accelerometer signal $a_{m}$ from the sensor frame into the world frame. After integrating the world frame IMU acceleration according to (3) and (4), there will be a non-zero residual velocity vector $v_{residual}$ as well as residual height $h_{residual}$. The idea of the proposed model is to express the accumulated velocity difference as a function of acceleration bias and HS vertical velocity error, and then correct for both. This is similar to taking the partial derivative of the velocity with respect to acceleration bias and HS vertical velocity error, then solving the equations to ensure that everything satisfies the zero residual velocity condition.

We use $v\left(k\right)$ and p(k) to express the velocity and position at sample k. The velocity starts at zero for each stride. Without the bias term and white noise term in (1), the velocity v(k) at current sample (k) is related to the prior velocity $v\left(k-1\right)$ and the current acceleration and rotation,

(6) $$v\left(k\right)=v\left(k-1\right)+\left[R\left(k\right){\cdot a}_{m}\left(k\right)+g\right]\cdot ∆t.$$

With the bias term in (3), the corrected velocity $v^{\prime }(k)$ at current sample (k) is

(7) $$v\prime \left(k\right)=v\prime \left(k-1\right)+[R\left(k\right)\cdot {(a}_{m}\left(k\right)-b)+g]\cdot ∆t.$$

We define the velocity difference $\delta v\left(k\right)$ as the difference of the velocity increment during sample (k−1) to (k) due to acceleration bias, and the accumulated velocity difference ∆v(k) up to sample k as the difference between $v\left(k\right)$ and $v^{\prime }(k)$:

(8) $$\left\{\begin{array}{c} \delta v\left(k\right)=\left[v\left(k\right)-v\left(k-1\right)\right]-{[v}^{\prime }\left(k\right)-v\prime (k-1)]\\ ∆v\left(k\right)=\sum_{i=1}^{k}\delta v(i)=v\left(k\right)-v\prime (k) \end{array}\right.$$

Similar to taking the partial derivative with respect to acceleration bias, we obtain an expression for $\delta v\left(k\right)$ as a function of acceleration bias by rearranging and subtracting (6) with (7):

(9) $$\delta v\left(k\right)=\left[v\left(k\right)-v\left(k-1\right)\right]-{[v}^{\prime }\left(k\right)-v\prime (k-1)]=R\left(k\right)\cdot b\cdot ∆t$$

Notice that this velocity difference is only dependent on the rotation matrix R(k). Recall that we assumed the rotation matrix R(k) is already computed. We further assume that the acceleration bias b and timestep ∆t are constant during each stride period. Then, the velocity difference $\delta v\left(k\right)$ in (9) is summed up over time to obtain the expression for accumulated velocity difference ∆v(k) from sample index 1 to sample index k as:

(10) $$\begin{array}{c} ∆v\left(k\right)=\sum_{i=1}^{k}\delta v\left(i\right)=\sum_{i=1}^{k}\left(R\left(i\right)\cdot b\cdot ∆t\right)\\ =\left(\sum_{i=1}^{k}R\left(i\right)\right)\cdot b\cdot ∆t=B_{v}\left(k\right)\cdot b\cdot ∆t. \end{array}$$

where the sum of rotation matrices is called $B_{v}(k)$

(11) $$B_{v}\left(k\right)=\sum_{i=1}^{k}R(i).$$

With this, we can represent the residual velocity ∆v(k) as a numerical function of acceleration bias $b={\left[b_{x},b_{y},b_{z}\right]}^{T}$. Note that $B_{v}(k)$ is no longer a rotation matrix; it is a general matrix made from the sum of several rotation matrices. 

Now consider the error that happens at heel strike. For each stride, the heel strike index $k_{hs}$ is detected as the first time the shoe touches the ground (see Section 5 for further discussion). Heel strike causes an impact opposite to the gravity direction as the foot lands on the ground. As discussed earlier, this impact might lead to the loss of information in the data because of limited sensor bandwidth or range. We model the loss of acceleration information as a discontinuity of velocity in the vertical direction, and call it heel strike (HS) vertical velocity error $∆v_{hs,z}$. It interacts with the system dynamics by adding this velocity impulse at the instant of heel strike,

(12) $$v_{z}\prime \left(k_{hs}\right)=v_{z}\left(k_{hs}\right)+∆v_{hs,z}$$

Combining the HS vertical velocity error $∆v_{hs,z}$ and velocity difference due to acceleration bias in (10), we can obtain the accumulated velocity difference $∆v\left(k\right)$ at sample k (for $k>k_{hs}$),

(13) $$\left[\begin{array}{c} \;{∆v}_{x}\left(k\right)\\ {∆v}_{y}\left(k\right)\\ {∆v}_{z}\left(k\right) \end{array}\right]=B_{v}\left(k\right)\left[\begin{array}{c} \;b_{x}\\ b_{y}\\ b_{z} \end{array}\right]∆t-\left[\begin{array}{c} 0\\ 0\\ ∆v_{hs,z} \end{array}\right].$$

To satisfy the zero residual velocity condition for each stride, the acceleration bias and HS vertical velocity error are computed such that the accumulated velocity difference will counter the non-zero residual velocity computed from raw data. The equation is simply a subtraction:

(14) $$∆v\left(N\right)=v\left(N\right)-v^{\prime }\left(N\right)=v_{residual}(N)-v_{residual}^{\prime }(N)=v_{residual}(N).$$

where N is the total number of samples in that stride, $v_{residual}(N)$ is the non-zero residual velocity integrated from the raw acceleration data up to sample N, and $v_{residual}^{\prime }(N)$ is the corrected residual velocity, which is set to zero to satisfy the ZUPT condition.

Therefore, the system of equations for satisfying the zero residual velocity condition with acceleration bias and heel strike velocity is

(15) $$\left[\begin{array}{c} \;v_{residual,x}\left(N\right)\\ v_{residual,y}\left(N\right)\\ v_{residual,z}\left(N\right) \end{array}\right]=\left[\begin{array}{ccc} B_{v,11}\left(N\right) & B_{v,12}\left(N\right) & B_{v,13}\left(N\right)\\ B_{v,21}\left(N\right) & B_{v,22}\left(N\right) & B_{v,23}\left(N\right)\\ B_{v,31}\left(N\right) & B_{v,32}\left(N\right) & B_{v,33}\left(N\right) \end{array}\right]\left[\begin{array}{c} \;b_{x}\\ b_{y}\\ b_{z} \end{array}\right]∆t-\left[\begin{array}{c} 0\\ 0\\ ∆v_{hs,z} \end{array}\right].$$

Note that Equation (15) alone has no unique solution, it needs to be combined with the zero height change condition described in the next section. 

### 3.2. Zero Height Change Assumption

Much of the time, walking does occur on level ground. Our method leverages this as a default assumption to ensure a zero height change in stride reconstruction. In a later step, the appropriateness of this assumption is evaluated to determine whether each stride was in fact on level ground, or instead on a ramp or on stairs; for these other cases, an alternative reconstruction is performed. 

Without the correction for acceleration bias b, at each sample k, the position p(k) is computed by Equation (16).

(16) $$p\left(k\right)=p\left(k-1\right)+v\left(k-1\right)\cdot ∆t+\frac{1}{2}\left[R\left(k\right)\cdot a_{m}\left(k\right)+g\right]\cdot {∆t}^{2}$$

With the correction for acceleration bias b, the corrected position $p^{\prime }(k)$ is computed as

(17) $$p^{\prime }(k)=p^{\prime }(k-1)+v^{\prime }(k-1)\cdot ∆t+\frac{1}{2}\left[R\left(k\right)\cdot {(a}_{m}\left(k\right)-b)+g\right]\cdot {∆t}^{2}.$$

Now we define the position difference δp(k) as the difference of position increment during sample (k−1) to (k) due to acceleration bias and accumulated position difference $∆p\left(k\right)$ up to sample k as the difference between p(k) and p′(k):

(18) $$\left\{\begin{array}{c} \delta p\left(k\right)=\left[p\left(k\right)-p\left(k-1\right)\right]-{[p}^{\prime }\left(k\right)-p\prime (k-1)]\\ ∆p\left(k\right)=\sum_{i=1}^{k}\delta p\left(i\right)=p\left(k\right)-p\prime (k) \end{array}\right..$$

Similar to taking a partial derivative with respect to acceleration bias, the position difference at time k is found by subtracting (17) from (16):

(19) $$\begin{array}{c} \delta p^{\prime }\left(k\right)=∆v\left(k-1\right)\cdot ∆t+\frac{1}{2}R\left(k\right)\cdot b\cdot {∆t}^{2}\\ ={[B}_{v}\left(k-1\right)+\frac{1}{2}R(k)]\cdot b\cdot {∆t}^{2}\\ ={[B}_{v}\left(k\right)-\frac{1}{2}R(k)]\cdot b\cdot {∆t}^{2}. \end{array}$$

Summing up (18) with the expression from (19) across samples, the accumulated position difference ∆p(k) up to sample k is:

(20) $$∆p\left(k\right)=\sum_{i=1}^{k}\delta p\left(i\right)=\sum_{i=1}^{k}[B_{v}\left(i\right)-\frac{1}{2}R\left(i\right)]\cdot b\cdot ∆t^{2}=B_{p}\left(k\right)\cdot b\cdot ∆t^{2}.$$

We use matrix $B_{p}(k)$ to represent the sum of matrix $B_{v}-\frac{1}{2}R$ from time 0 to sample k:

(21) $$B_{p}\left(k\right)=\sum_{i=1}^{k}B_{v}\left(i\right)-\frac{1}{2}R\left(i\right).$$

The HS vertical velocity error will also contribute to the position difference. The velocity impulse $∆v_{hs,z}$ acts on the vertical velocity at time $k_{hs}$. Propagating its effect on velocity to the end of the stride at sample N, the position difference due to HS vertical velocity error will be

(22) $$\left[\begin{array}{c} \;{∆p}_{x}\left(N\right)\\ {∆p}_{y}\left(N\right)\\ {∆p}_{z}\left(N\right) \end{array}\right]=-\left[\begin{array}{c} 0\\ 0\\ ∆v_{hs,z} \end{array}\right]\left(N-k_{hs}\right)∆t.$$

Since level-ground walking means zero changes in height, only the position component in the vertical direction needs to be considered in (20). Expanding the matrix form in (20) and keeping the third row, we obtain

(23) $$∆p_{z}\left(n\right)=\left[B_{p,31},B_{p,32},B_{p,33}\right]{\left[b_{x},b_{y},b_{z}\right]}^{T}{∆t}^{2}.$$

Combining (22) and (23), we have the following equation to compute accumulated height difference $∆p_{z}\left(N\right)$ due to acceleration bias and HS vertical velocity error.

(24) $$∆p_{z}\left(N\right)=\left[B_{p,31},B_{p,32},B_{p,33}\right]\left[\begin{array}{c} b_{x}\\ b_{y}\\ b_{z} \end{array}\right]{∆t}^{2}-∆v_{hs,z}\cdot \left(N-k_{hs}\right)\cdot ∆t$$

To satisfy the zero height change condition, acceleration bias and HS vertical velocity error are computed such that they will counter the non-zero residual height. Similar to Equation (14), the residual height is connected to the model:

(25) $$∆p\left(N\right)=p\left(N\right)-p^{\prime }\left(N\right)=h_{residual}(N)-h_{residual}^{\prime }(N)=h_{residual}(N).$$

where N is the number of samples for each specific stride, $h_{residual}(N)$ is the non-zero residual height double integrated from raw acceleration data, and $h_{residual}^{\prime }(N)$ is the corrected residual height and should be zero to satisfy zero height change condition.

### 3.3. Matrix Model

Combining the accumulated velocity difference Equations (13) and the accumulated height difference Equation (24) together, the system of equations connecting the acceleration bias and heel strike velocity error with velocity difference and height difference at the same time is formulated:

(26) $$\left[\begin{array}{c} \;{∆v}_{x}\left(N\right)\\ {∆v}_{y}\left(N\right)\\ {∆v}_{z}\left(N\right)\\ ∆p_{z}\left(N\right) \end{array}\right]=\left[\begin{array}{cccc} B_{v,11}\left(N\right) & B_{v,12}\left(N\right) & B_{v,13}\left(N\right) & 0\\ B_{v,21}\left(N\right) & B_{v,22}\left(N\right) & B_{v,23}\left(N\right) & 0\\ B_{v,31}\left(N\right) & B_{v,32}\left(N\right) & B_{v,33}\left(N\right) & -1/∆t\\ B_{p,31}\left(N\right)∆t & B_{p,32}\left(N\right)∆t & B_{p,33}\left(N\right)∆t & -\left(N-k_{hs}\right) \end{array}\right]\left[\begin{array}{c} \;b_{x}\\ b_{y}\\ b_{z}\\ ∆v_{hs,z} \end{array}\right]∆t\;$$

**Figure 1:**
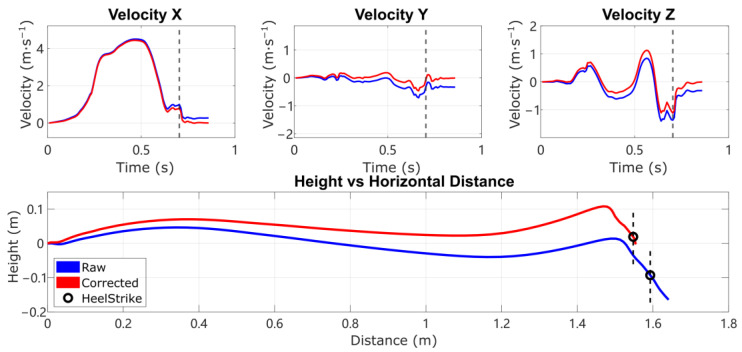
Three-axis velocity and horizontal trajectory in the sagittal plane (x forward, z up), with the instant of heel strike noted as the dashed line and circle. The proposed method de-drifts the residual non-zero velocity and non-zero height change by estimating acceleration bias and HS vertical velocity error.

When a heel strike event is detected and the movement is level-ground walking, we can solve for a unique set of ${\left[b_{x},b_{y},b_{z},∆v_{hs,z}\right]}^{T}$ as in (27) such that they will compensate for the non-zero residual velocity and non-zero height change as shown in Figure 1.

(27) $$\left[\begin{array}{c} \;{∆v}_{x}\left(N\right)\\ {∆v}_{y}\left(N\right)\\ {∆v}_{z}\left(N\right)\\ ∆p_{z}\left(N\right) \end{array}\right]=\left[\begin{array}{c} \;v_{residual,x}\left(N\right)\\ v_{residual,y}\left(N\right)\\ v_{residual,z}\left(N\right)\\ h_{residual,z}\left(N\right) \end{array}\right]$$

Putting (26) and (27) together yields the system of equations that guarantee any assumed residual velocity and height change,

(28) $$\left[\begin{array}{c} \;v_{residual,x}\left(N\right)\\ v_{residual,y}\left(N\right)\\ v_{residual,z}\left(N\right)\\ h_{residual,z}\left(N\right) \end{array}\right]=\left[\begin{array}{cccc} B_{v,11}\left(N\right) & B_{v,12}\left(N\right) & B_{v,13}\left(N\right) & 0\\ B_{v,21}\left(N\right) & B_{v,22}\left(N\right) & B_{v,23}\left(N\right) & 0\\ B_{v,31}\left(N\right) & B_{v,32}\left(N\right) & B_{v,33}\left(N\right) & -1/∆t\\ B_{p,31}\left(N\right)∆t & B_{p,32}\left(N\right)∆t & B_{p,33}\left(N\right)∆t & -\left(N-k_{hs}\right) \end{array}\right]\left[\begin{array}{c} \;b_{x}\\ b_{y}\\ b_{z}\\ ∆v_{hs,z} \end{array}\right]∆t,$$

where the left-hand side is the residual velocity and height integrated from raw acceleration data for level-ground walking without slipping. Matrix $B_{v}(N)$ and $B_{p}(N)$ can be computed iteratively using the following equations (where $B_{v}\left(0\right)$ and $B_{p}\left(0\right)$ are both zero matrices):

(29) $$\left\{\begin{array}{c} B_{v}\left(k\right)=B_{v}\left(k-1\right)+R\left(k\right)\\ B_{p}\left(k\right)=B_{p}\left(k-1\right)+B_{v}\left(k\right)-\frac{1}{2}R\left(k\right) \end{array}\right.$$

## 4. Terrain Determination

Walking on ramps and stairs involves different gait patterns compared with level-ground walking. These alternative terrains are common in both indoor and outdoor environments, yet they impose extra challenges for people with mobility interventions such as orthosis and prosthesis users [57,58]. To understand walking performance on ramps and stairs in real-world settings, it is essential to both identify the ramps and stairs in the dataset and to reconstruct them accurately.

However, without any assumption or external information about the terrain, both the KF-based methods [19,26] and integration-based method [11] suffer from drift in height. One can force the zero height change condition for level-ground walking, but then the question is how to determine if people are walking on level ground or not, so as to determine when to apply or not apply the level-ground walking assumption. Much work has been conducted to detect ramps and stairs activities using different combinations of wearable sensors signals [59], including motion data from IMUs, muscle activity from electromyographic sensors (EMG), ground reaction force/moment from pressure insoles and prosthetic pylons, etc. However, when it comes to practical clinical use, a trade-off has to be considered regarding accuracy/performance and availability, as some types of sensors are hard to deploy outside the laboratory environment. This section derives a new approach to achieve both terrain determination with high accuracy and improved accuracy of reconstruction using only a single foot-mounted IMU. 

Equation (27) is derived by imposing both a zero velocity assumption and zero height change assumption at the same time. These assumptions are only correct for level-ground walking. However, this equation can still be solved for data from ramp and stair walking, and by examining the solution vector $\left[b_{x},b_{y},b_{z},∆v_{hs}\right]$, it can be determined whether each stride is indeed walking on level ground, or alternatively on a ramp or stair.

To achieve this ramps and stairs determination, for each stride i, the first step is to double integrate the raw acceleration data in the world frame to obtain residual velocity and residual position, then apply Equation (27) to obtain the solution vector ${\left[b_{x}\left(i\right),b_{y}\left(i\right),b_{z}\left(i\right),{∆v}_{hs}\left(i\right)\right]}^{T}$ and collect these solutions into a stride-by-stride array.

**Figure 2:**
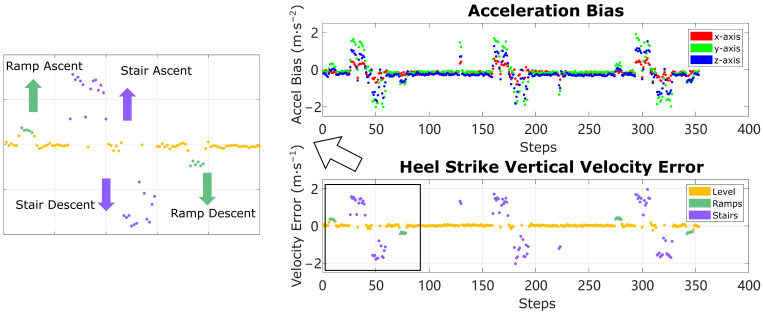
Acceleration bias and HS vertical velocity error among all strides in an example trial. All large deviations in both figures belong to ramps/stairs strides. Additionally, the direction of deviation of HS vertical velocity error indicates the ascent/descent direction on ramps and stairs.

Figure 2 shows the solution vector ${\left[b_{x},b_{y},b_{z},∆v_{hs}\right]}^{T}$ across all strides on a 7-min trial with ramps, stairs, and level-ground walking. The top figure shows each component of the acceleration bias solution, and the bottom figure shows the heel strike vertical velocity error, with a magnified view on the left.

Any obvious deviation from the average value of the solution vector belongs to the strides on ramps/stairs. To remove the baseline effect of level-ground walking, in each trial the mean of the 2nd and 3rd quartile of the acceleration bias and HS vertical velocity error data are subtracted from those respective signals, then two different thresholds are applied to separate level ground/ramps/stairs. By empirical tuning for a proper threshold that encourages the most separation, level ground, stairs, and ramps can be cleanly distinguished as long as the effect of sensor noise, errors in orientation estimation, and walking pattern (for example, magnitude of heel strike) will not blur the effect of violating the level-ground condition, which is true for most of our trials in Section 6. In practice, the HS vertical velocity error is used to separate level ground vs. ramps vs. stairs. The acceleration bias is only used to separate non-stairs vs. stairs. More details on choosing thresholds are discussed in Section 6 Experiments and Section 7 Discussion. As shown later in the paper, the ramp in the test data set has only 3.7-degree inclination angle, yet this method accurately captures the deviation in solution vectors. When walking on ramps with steeper inclination angles, the solution vector should only become easier to separate. 

The magnitude of deviation is significantly larger on stairs than on ramps, enabling each category to be separated individually. Moreover, the sign of the HS vertical velocity error corresponds to the direction of height change, either going up or down. This behavior makes sense because when Equation (27) is used to force a stride with height change to be reconstructed as level-ground walking, the algorithm will compute a large acceleration bias and velocity error to compensate for the height change. Since the heel strike velocity error is expressed in the world frame, an HS vertical velocity error $∆v_{hs,z}$ in the same direction as true height change in the stride is found, so that it can eliminate the height change. In contrast, the acceleration bias b is expressed in the IMU local frame, the direction of which is less intuitive. Both acceleration bias and HS vertical velocity error show stable distribution among the strides on level ground, which confirms that specific patterns exist in steady level-ground walking. The fluctuation of the solution vector is a result of the unmodeled sensor noise or imperfections in the orientation estimation. 

The proposed method is good at separating level-ground walking from non-level-ground walking. One challenge in practice is that the boundary between ramps and stairs can be vague in terms of only height change. For example, the first stride on stairs often only rises one stairstep, resulting in a lower height change compared with the strides in the middle of the stairs that cover two stairsteps. To improve the detection accuracy, a final check on the detection results can be performed using the following conditions:

(1) Strides on ramps have larger forward distance and less height change than strides on stairs.
(2) Strides on ramps or strides on stairs are usually consecutive.

The forward distance can be extracted from the trajectory reconstructed from the raw acceleration data which are used to compute $v_{residual}$ and $h_{residual}$; thus, there is no need to recompute anything. 

Occasionally, heel strike events are not detected for some strides. An average heel strike timing is assigned to the undetected stride only for the purpose of ramps and stairs detection, not for the trajectory reconstruction in Section 5.

The terrain determination method is summarized as the following Algorithm 1:
**Algorithm 1**: Terrain Determination.**Input:** acceleration data, orientation data, ZUPT index, heel strike index. **for** each stride j **do**:  $\mathrm{double}\;\mathrm{integrate}\;\mathrm{world}-\mathrm{frame}\;\mathrm{acceleration}\;\mathrm{to}\;\mathrm{obtain}\;v_{residual}$$\;\mathrm{and}\;h_{residual}$  **if**$\;\mathrm{no}\;k_{hs}$ **do**    $\mathrm{assign}\;\mathrm{an}\;\mathrm{average}\;\mathrm{value}\;\mathrm{to}\;k_{hs}$  **end if**  solve Equation (27) to obtain ${\left[b_{x}\left(j\right),b_{y}\left(j\right),b_{z}\left(j\right),{∆v}_{hs,z}\left(j\right)\right]}^{T}$ **end for** obtain mean $∆v_{mean}=$$\;\mathrm{mean}\;\mathrm{of}\;2\mathrm{nd}\;\mathrm{and}\;3\mathrm{rd}\;\mathrm{quartile}\;\mathrm{of}\;∆v_{hs,z}$ obtain mean $b_{mean}=$ mean of 2nd and 3rd quartile of b **for** each stride j **do**:  **if** $\left|{∆v}_{hs,z}\left(j\right)-∆v_{mean}\right|>$ V_THRESHOLD_RAMP **do**    RampStairIndicator(j) = RAMP  **end if**  **if** $\left|b(j)-b_{mean}\right|>$$\;B\_\mathrm{THRESHOLD}\_\mathrm{STAIR}\;\mathrm{or}\;\left|{∆v}_{hs,z}\left(j\right)-∆v_{mean}\right|>$ V_THRESHOLD_STAIR **do**    RampStairIndicator(j) = STAIR  **end if**  $\mathrm{direction}=\mathrm{sign}({∆v}_{hs,z}(j)$) **end for** **for** strides that are ramps/stairs **do**  clean wrong determination by forward distance and height change criterion  clean wrong determination by consecutive ramps/stairs criterion **end if**

## 5. Reconstruction Method

The core idea of this paper is to simplify the error states to acceleration bias and HS vertical velocity error, build a new model in Section 3 to estimate them, and apply this model to develop a new form of integration-based motion reconstruction method to reduce the drift and error during reconstruction. The proposed method is different from the widely used ZUPT-based integration reconstruction because it does not simply assume the drift is linear; instead, it views the error from the perspective of an IMU error model—specifically, discrete loss of information in the heel strike impact. The method is also different from ESKF reconstruction because it is a deterministic approach in computing the bias and error terms instead of a probabilistic approach. 

### 5.1. Preprocessing

The preprocessing step includes computing the orientation information from raw IMU data, then extracting basic gait information such as heel strike index and ZUPT index, which will be used in reconstruction. There are several steps:

(1) The orientation of the IMU sensor can be obtained from any suitable algorithm, for example, direct quaternion integration with gravity correction during stance phase [1] or Kalman Filter for only orientation if the goal is to reconstruct IMU motion, or even the orientation from the ESKF-based PDR if the goal is to improve the reconstruction results. Results presented here use the orientation quaternion from the ESKF method, because the orientation itself is not affected by HS impact as much as position and velocity. We also provide results using orientation data without Kalman Filter (i.e., using direct quaternion integration of gyro data) in Appendix A. 

(2) The orientation data are used to transform the acceleration data from the IMU local frame to the global frame. 

(3) The heel strike detector will look for the first high peak in the difference of vertical acceleration [18,60,61]. 

(4) The ZUPT index indicates whether the foot is in the “foot-flat” phase (i.e., heel and forefoot both contacting the ground), so that the zero velocity assumption is satisfied. The ZUPT index is a Boolean defined as “true” when both acceleration and angular velocity indicate low movement:

$$ZUPT\left(k\right)=\left|\left\|\begin{array}{c} a\left(k\right) \end{array}\right\|-g\right|<T_{a}\;\&\;\left\|\begin{array}{c} \omega \left(k\right) \end{array}\right\|<T_{\omega }$$

$T_{a},T_{\omega }$ are the thresholds for acceleration and angular velocity. This defines a finite period of time in each stride, similar to the ZUPT index in most PDR literature. The ZUPT index is subsequently smoothed to remove any small stance phase and high motion periods that might be wrong, and then each contiguous period of the ZUPT index is reduced to its central portion by trimming off the ends, to ensure only the most stationary period is identified. Throughout the ZUPT period, the velocity is held as zero to eliminate stance phase drift and thereby reduce displacement errors further. In the limit, the ZUPT period can be reduced to a single index. 

(5) Use Algorithm 1 to obtain the ramps and stairs indicator. The ramps and stairs indicator will determine how each stride is reconstructed based on whether or not the zero height change condition applies. 

### 5.2. Reconstruction

For each stride, the reconstruction algorithm is also run on the raw IMU data first to obtain residual velocity $v_{residual}$ and residual height $h_{residual}$, followed by the model developed in Section 3. When the stride is on level ground, both zero velocity and zero height change assumptions are applied to solve for a unique solution of $\left[b_{x},b_{y},b_{z},∆v_{hs,z}\right]$ according to Equation (27). When the stride is on ramps and stairs, the zero height change assumption does not apply, so instead the reconstruction reverts to a traditional ZUPT algorithm through Equation (15), assuming a HS vertical velocity error of zero to generate a unique solution. This is necessary because there is no fourth equation to separate the effects of b and $∆v_{hs}$. 

After determining $\left[b_{x}\left(j\right),b_{y}\left(j\right),b_{z}\left(j\right),{∆v}_{hs,z}\left(j\right)\right]$ for the stride (j), the acceleration is corrected by subtracting the acceleration bias, and is then rotated into the world frame and double integrated into v(k) and p(k). When the current time is the heel strike moment, the HS vertical velocity error $∆v_{hs,z}$ is added to v(k) according to (12) (having no effect on ramps and stairs when $∆v_{hs,z}$ is set to zero).

Considering that there are unmodeled noise and bias in the system and that sensors could have different levels of noise, we introduce an extra correction parameter C to account for the uncertainty of noise. C will decide the portion of residual velocity that is corrected using the error model. For better, more accurate IMU sensors, C can be set close to 1. For sensors with higher noise levels, C should be set to a lower value, for example 0.9. The intuition for parameter C is that, for better sensors, the unmodeled noise contributes less to the residual velocity and height; thus, we can attribute all residual velocity to acceleration bias and HS vertical velocity error (C = 1). For noisier or less accurate IMU data, the unmodeled random noise contributes a non-negligible portion of the residual velocity and height; thus, not all of the error estimated by the error model should be used for a correction. The reconstruction algorithm is shown as the Algorithm 2 below.
**Algorithm 2**: Trajectory Reconstruction.**Input:** acceleration data, orientation data, ZUPT index, heel strike index. Use Algorithm 1 to detect all ramps and stairs. Convert raw acceleration data to global acceleration data accel(k) **for** each stride j **do**  $\mathrm{double}\;\mathrm{integrate}\;\mathrm{the}\;\mathrm{raw}\;\mathrm{acceleration}\;\mathrm{to}\;\mathrm{obtain}\;v_{residual}$$\;\mathrm{and}\;h_{residual}$  $\mathrm{scale}\;v_{residual}$ with parameter C  **if** RampStairIndicator(j) = false **do**    solve Equation (27) to obtain ${\left[b_{x}\left(j\right),b_{y}\left(j\right),b_{z}\left(j\right),∆v_{hs,z}\left(j\right)\right]}^{T}$  **else do**    solve Equation (15) to obtain ${\left[b_{x}\left(j\right),b_{y}\left(j\right),b_{z}\left(j\right)\right]}^{T}$    $\mathrm{set}\;∆v_{hs,z}=0$  **end if**  $accel\left(1..N\right)$
*=*
$accel\left(1..N\right)$
*−*
${\left[b_{x}\left(j\right),b_{y}\left(j\right),b_{z}\left(ij\right)\right]}^{T}$  ***for***
*k = 1:N **do***    $v\left(k\right)=v\left(k-1\right)+accel\left(k\right)*∆t$  **if**$k=k_{hs}$ **do**    $v\left(k\right)=v\left(k\right)+∆v_{hs,z}(j)$  **end if**  $p\left(k\right)=p\left(k\right)+p\left(k\right)*∆t+\frac{1}{2}accel\left(k\right){∆t}^{2}$  **end for** **end for**

## 6. Experiments

The proposed algorithm was tested for 15 trial records (duration 8.01±1.63 min) across both feet of 6 subjects (4 female, 2 male, age 27±3 years old, height 1.74 ± 0.07 m, weight 83.1± 14.4 kg), including 5 able-bodied subjects and 1 person with unilateral transtibial amputation. Participants gave their written informed consent according to procedure approved by the University of Wisconsin–Madison Health Sciences Institutional Review Board (HS-2017-0678), in accordance with the Declaration of Helsinki. We used Opal wearable IMUs (APDM Wearable Technologies, OR, USA) and attached them to the distal portion of the shoelaces with a tight-fitting fabricated pouch. These sensors collect gyroscope and accelerometer data at 128 Hz. Although the sensor measures magnetic data and barometric pressure data at the same time, the algorithm does not use these signals. In the testing, the subjects walked a route in an academic building three times, including long periods of level walking, short ramps, a short stairway, and a long stairway to the next floor of the building. They visited the long stairway exactly 3 times but visited the short ramps and short stairway in random order and random number of times. The ramp has a 3.7-degree incline angle, and both the ramps and the short stairs have a height of 0.6 m; the main staircase is 4.5 m tall with a landing in the middle. The height of individual stairs in the short staircase is 0.15 m, and in the main staircase 0.18 m. The numbers of stairs are 4 for the short stairway and 25 for the long stairway. These characteristics of the building terrain define ground-truth for quantifying the accuracy of the results.

After collecting the dataset, the IMU trajectory was reconstructed using Algorithm 2, which contains the usage of Algorithm 1 to detect all the ramps and stairs in the dataset. For the algorithm settings, the ZUPT period was reduced to the central 80 percent of each contiguous period of ZUPT index and the correction parameter was set to 1 (full correction). Results of the reconstruction were evaluated by the height error at the beginning and end of each terrain segment, according to the ground truth of building geometry. For level ground, the reference height change was zero; for ramps and the short stairway, the reference height is 0.6 m; for the long stairway, the reference height is 4.5 m. The results from the proposed method were compared against an ESKF reconstruction [4,15,28] as well as integration reconstruction with ZUPT that de-drifts error linearly [1,11,13,14]. Note that both the proposed method and integration reconstruction with ZUPT are forms of integration-based reconstruction.

### 6.1. Results: Terrain Determination

**Figure 3:**
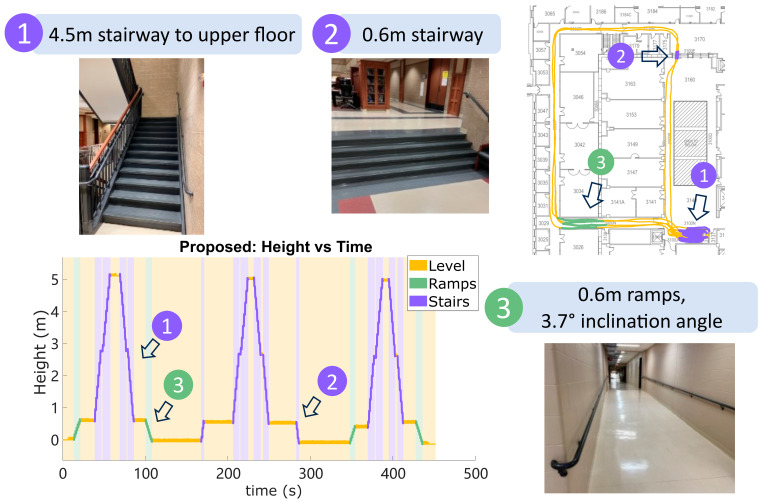
The example reconstruction results from one trial with 3 long stairways, 2 short stairways, and 4 ramps. The whole path includes a 0.6 m ramp with 3.7-degree inclination angle, 4.5 m stairs to the upper floor, and short 0.6 m stairs. The horizontal trajectory is plotted on the true floor plan of the Mechanical Engineering Building 3rd floor. The Height vs. Time figure labels 3 terrains with unique colors. All the strides on the turn between long stairs ascent and descent are correctly detected as level ground.

**Figure 4:**
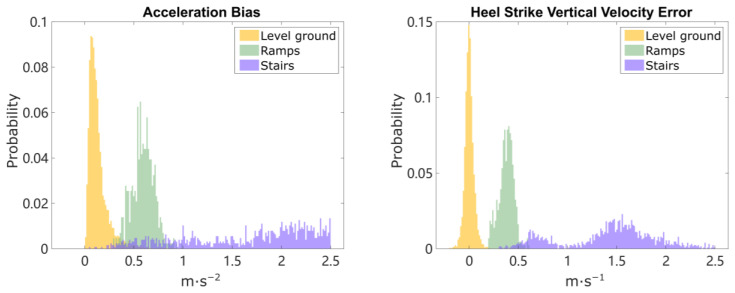
Histogram of acceleration bias (magnitude of b) and heel strike vertical velocity error (magnitude of the $∆v_{hs,z}$) among all 15 trials, including 4308 strides on level ground, 433 strides on ramps and 1268 strides on stairs. The histogram is normalized so that the probability of strides sums up to 1 for each terrain type, and the mean of the 2nd and 3rd quartiles of $∆v_{hs,z}$ is subtracted to remove base effect on level ground. A clear separation is shown on HS vertical velocity error between level ground vs. non-level ground strides.

Figure 3 shows an example reconstruction of the algorithm. The results of terrain determination and the following height error were compared against the manual segmentation of the known structured indoor environment. All the stairs and ramps segments are correctly detected and the total height error at the end point is less than 0.2 m. It is worth mentioning that the short turning space at the landing in the middle of the staircase is also correctly detected, showing the fine resolution of the terrain determination. Terrain determination Algorithm 1 was run on all 15 trials, which counted 6009 strides in total. The detected number of strides on ramps was 433 and the number of strides on stairs was 1268. The determination results are summarized in Table 1. The overall accuracy for all three terrains is 99.7%. The accuracy of each terrain is 99.9% for level ground, 96.0% for ramps, and 100% for stairs. Out of the 18 strides on ramps that were misclassified as level ground, 17 were the transition strides on the edge of ramps. Those transition strides are hard to detect [51] as they have shorter height change that approaches the limit of the proposed fixed-thresholds method, meaning some strides on ramps are determined as level ground due to minimal height change. The magnitude of the computed acceleration bias and the heel strike vertical velocity error are plotted in normalized histograms in Figure 4. Three clusters are formed, for level ground, ramps, and stairs. In the histogram for HS vertical velocity error, a clear separation between the level ground and ramps can be observed. The empirical way of finding potential thresholds can be achieved by examining the outputs as shown in Figure 2 and choosing the velocity thresholds that encourage the most separation between level ground/ramps and ramps/stairs. The thresholds remain the same for all 15 trials, with the thresholds V_THRESHOLD_RAMP set to 0.2 m/s, V_THRESHOLD_STAIR set to 1 m/s, and B_THRESHOLD_STAIR set to $1.2\;m/s^{2}$. The height change and forward swing distance for separating ramps and stairs are 0.3 m and 1 m.

### 6.2. Reduced Height Error

**Figure 5:**
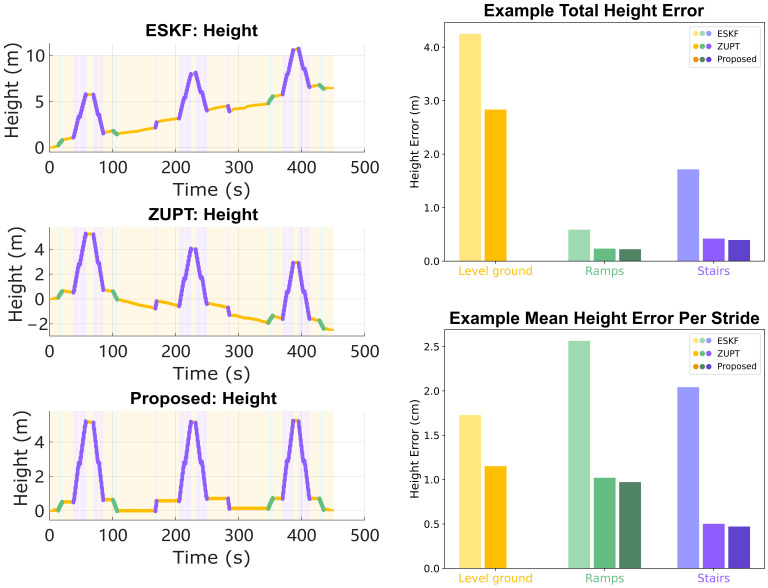
Height trajectory and error analysis of the example reconstruction result. Left: height–time plots reconstructed using three methods: ESKF, ZUPT, and proposed. Right: Total height error and mean height error per strides on 3 terrains. The proposed method removes 99% of the error on level ground while achieving similar performance on ramps and stairs.

**Figure 6:**
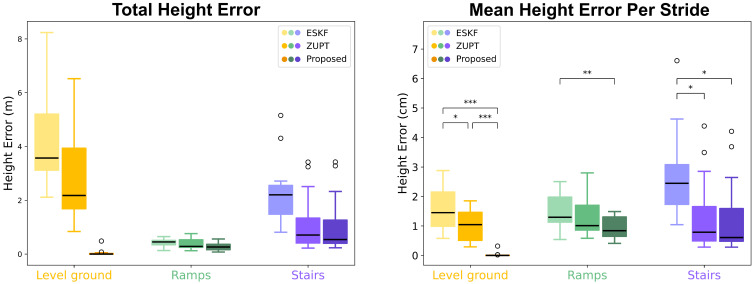
Overall error analysis for all 15 trials. The proposed method removes 99% of the error on level ground while achieving slightly improved performance on ramps and stairs (*t*-test values: * *p* < 0.05; ** *p* < 0.01; *** *p* < 0.001).

The accuracy of height information can be characterized by computing the accumulated height error on each terrain (Figure 5 and Figure 6). The average height error on a terrain is the total height error divided by the number of strides on that terrain. The mean height errors per stride are 0.03±0.08 cm on level ground, 0.95±0.37 cm on ramps, and 1.27±1.22 cm on stairs. The statistical significance of the differences in mean height error per stride from the three methods (ESKF vs. ZUPT, ESKF vs. Proposed, ZUPT vs. Proposed) are computed using two-tail two-sample Student’s t-tests on each terrain; the results are presented in Figure 6. The proposed method shows greatly improved accuracy on level ground in terms of height error because it explicitly imposes the zero height change conditions. It demonstrates similar or slightly better accuracy on ramps and stairs compared with the ZUPT method. 

### 6.3. Accuracy

**Figure 7:**
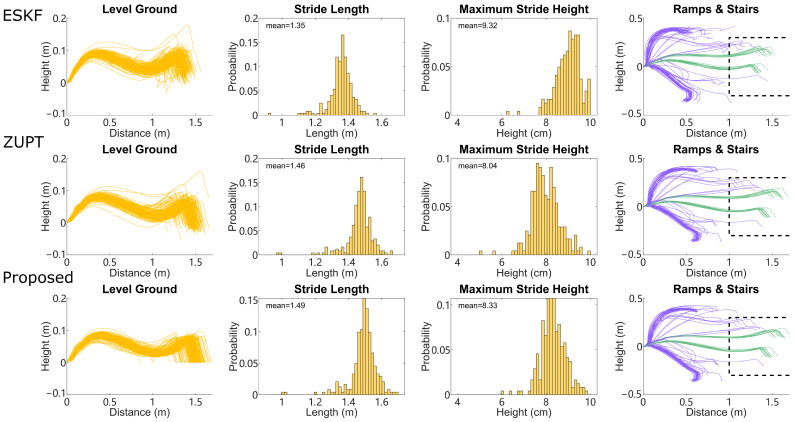
Stride lengths and stride heights of proposed method compared with the ZUPT method and ESKF method, in one example trial. (**Left**): sagittal view of reconstructed strides for level walking using each method. (**Center-left**): distribution of stride length. (**Center-right**): distribution of maximum IMU height during each stride. (**Right**): sagittal view of reconstructed strides for ramps (green) and stairs (purple), dash line indicating the kinematic boundary for separating ramps vs. stairs by trajectory. Further separation is achieved by the consecutive ramps/stairs criterion.

The accuracy of the reconstruction from the proposed method is compared to the other two methods on a stride-by-stride basis in Figure 7, where we compute the stride length and maximum relative stride height from all three methods for the same stride on level-ground walking. Strides from the compared methods have nonzero residual height, because in the compared methods themselves, terrains cannot be determined accurately, and thus cannot be applied with the zero height change condition confidently. As shown in Figure 7, both integration-based methods have similar stride length and maximum stride height in terms of mean and distribution. The ESKF method shows slightly shorter stride length and slightly higher maximum stride height. Figure 7 also shows reconstructed IMU trajectories on stairs and ramps, which are clearly delineated into rising and falling trajectories. Both stairs and ramps have substantial variation, depending in part on the kinematics of each specific stride, such as foot placement at the very beginning or end of a ramp, or stepping onto the first vs. second step of a staircase from different approach distances.

## 7. Discussion 

### 7.1. Implementation of Terrain Determination

This paper proposed an integration-based Pedestrian Dead Reckoning (PDR) algorithm that can achieve accurate terrain determination and reconstruction of IMU trajectory with improved accuracy in height estimation using a single foot-mounted IMU. The key advancement is the introduction of a kinematic model that estimates both acceleration bias and heel strike vertical velocity error by leveraging the zero velocity condition and zero height change condition on level-ground walking. By applying the level ground assumption to strides from all terrains, the algorithm generates large differences in model outputs for strides on ramps and stairs due to violation of the level-ground condition. With a proper threshold, these differences, specifically the HS vertical velocity error, yield a clear separation of level-ground walking from non-level-ground walking, which indicates that the proposed method is sensitive enough to separate the non-level ground from level ground. Considering the fact that the ramp incline is only 3.7 degrees, this level of separation is already very good. However, depending on the sensor capability, orientation estimation accuracy, and walking pattern, the separation could be sub-optimal as the effect of errors mentioned above surpass the effect of the violation level-ground condition. In this situation, the strides connecting level ground with non-level-ground terrain will be less distinct; therefore, it is important to use a good-quality sensor to ensure good results. 

The most critical threshold is V_THRESHOLD_RAMP that separates level ground from ramps. The height change on ramps can be quite small, and in such cases it will not create a large deviation in model outputs. If the threshold value is too small, false positive detection may happen on ramps; if it is too high, ramps may be ignored. In our dataset, a value between 0.2 m/s to 0.22 m/s generated robust results on all 15 trials. If the application allows tuning to individual subjects, the optimal thresholds may vary across a larger range, thus being more robust to different thresholds. One of the prosthetic foot trials is slightly sensitive to this threshold, because of the increased error in both velocity and orientation estimation induced by significantly larger heel strike impact (see below). 

The thresholds for stair detection, V_THRESHOLD_STAIR and B_THRESHOLD_STAIR, are less sensitive as the height change on the staircase is larger. Though the region of HS vertical velocity errors between the clusters of ramps and stairs is slightly blended, as shown in Figure 4, further separation of ramps from stairs is achieved using a rectangular kinematic boundary (Figure 7 right column) and a consecutive ramps/stairs criterion. Other available strategies include using an elliptical boundary on IMU trajectory [51], or using stance phase pitch angle of the foot to distinguish ramps from both stairs and level ground [19,50]. The proposed method can also be applied to any framework where a limited number of IMU sensors are available for ramp and stair detection. Note that the proposed method is tested on structured indoor environments, while in real-world settings, the baseline level-ground effect may change as the walking patterns change due to factors like hardness of the ground, fatigue during long bouts of walking, or different footwear. Uneven terrain may also add more difficulty because zero height change becomes an approximation and no longer holds true.

### 7.2. Physically Meaningful De-Drifting Leads to Improved Accuracy

**Figure 8:**
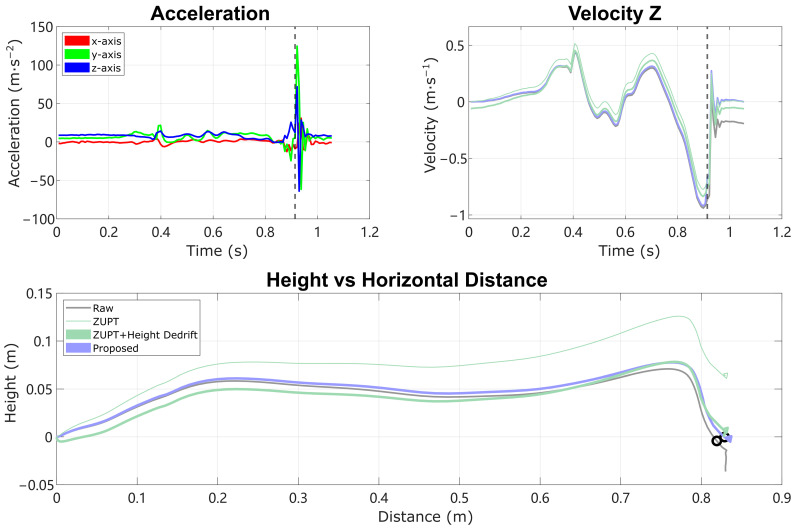
Reconstruction of an example stride with large heel strike impact, comparing trajectory reconstructed from Raw integration, traditional ZUPT, ZUPT combined with linear height de-drifting (modeled as a constant bias in velocity z), and the Proposed method. (**Top left**) plot is the raw acceleration data in the IMU local frame. (**Top right**) plot is the vertical velocity in the world frame. (**Bottom plot**) is the IMU trajectory in the sagittal plane (x forward, z up), with the instance of heel strike noted as a dashed line and circle. Note the flaws in the comparison methods: Raw integration leaves drift during the following stance phase; traditional ZUPT generates substantial height error; and ZUPT with linear height de-drifting yields negative height at the beginning of the movement and reduced height during forward swing.

After determining the terrain type, the reconstruction is performed again, applying the full model or reduced model to estimate the acceleration bias and (for level ground only) the HS vertical velocity error, to remove the drift in the IMU trajectory reconstruction. In human subject experiments, the reconstruction results demonstrate improved accuracy in the vertical direction on all three terrains compared with ESKF-based reconstruction and traditional integration reconstruction with ZUPT. Some past work has shown that linear detrending can eliminate the remaining height error [10,12], but this can also generate unrealistic stance-phase foot movements, leading to efforts to detrend with other curves such as sigmoidal fits [3]. Moreover, these are purely empirical and are not based on known physical error sources. A comparison of the reconstruction of an example real-world stride on level ground with large heel strike impact is shown in Figure 8. For the ZUPT method, the zero height change condition is enforced by linear de-drifting the residual velocity, which is reflected as a fixed velocity offset in vertical velocity [10]. The final trajectory of the ZUPT method demonstrates unrealistic negative height at the beginning of the motion and reduced height throughout the forward swing. The proposed method is conceptually better because it de-drifts the velocity and position with a physically meaningful model of impact of the foot with the ground, instead of assuming linear or otherwise parameterized drift. Thus, the height trajectory is accurate not only at the final position, but also throughout the whole swing phase. This approach can therefore improve the metrics of the swing phase movement that depend on accurate vertical movement.

It is not surprising to see that the proposed method and ZUPT demonstrate similar stride length and height for level-ground walking and similar per-stride error on ramps and stairs (Figure 7), as they are both integration-based reconstruction methods and the proposed method does not use any additional information for these terrains. Compared with the ESKF method, both integration-based reconstructions show higher accuracy on all terrains. That being said, in practice, the strength of the filter-based method lies on the sensor fusion side (for IMU orientation), not the reconstruction accuracy side. The orientation of the IMU sensor at each moment is assumed to be obtained by any orientation estimation methods as discussed in Section 2 from gyroscope and accelerometer data. In Appendix A, we provide the results using a non-Kalman Filter-based orientation estimation approach. To mitigate the common heading drift issue, it is also possible to improve the heading estimation by enabling magnetometer data and using other sensor fusion algorithms [56].

### 7.3. Exploring and Quantifying Heel Strike Error

The estimation and correction of impact-induced HS vertical velocity error is the new advancement of the proposed kinematic model. As observed in Figure 4, the distribution of HS vertical velocity error on level ground and ramps seems to follow a normal distribution, while on stairs it does not show a normal distribution. The distribution for stairs may be bimodal, reflecting one-stair and two-stair strides. It is important to note that the HS vertical velocity error computed for ramps and stairs does not have the same physical meaning as in the level-ground condition, because the terrain determination forces all strides to satisfy the zero height change assumption. It is only useful in the sense of determining terrains. However, an understanding of the probabilistic distribution of the parameters may be useful in supporting future work.

**Figure 9:**
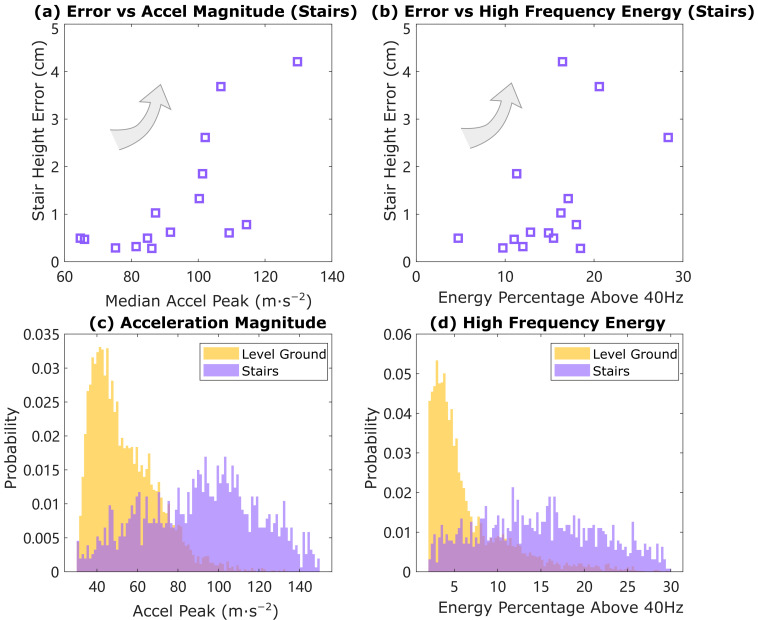
The magnitude of heel strike is quantified by the maximum peak acceleration magnitude and acceleration energy percentage above 40 Hz in accelerometer signals. (**Top**): median peak and energy percentage with respect to mean height error on stairs for all 15 trials. As an observation, the mean height error on stairs increases as both peak acceleration and energy percentage increases. (**Bottom**): peak and energy percentage for all strides on level ground and stairs. The distribution of both acceleration peak and energy percentage differs on the two terrains.

The HS vertical velocity error turns out to be different for each subject depending on the way they walk; for example, how they land on heel strike or how heavily or gently they walk on stairs. We characterized the high-frequency impact in the acceleration data (mainly due to heel strike) by computing the peak value of acceleration in each stride and the high-frequency acceleration signal energy (percentage of total energy in the power spectrum between 40 Hz and 64 Hz, through the whole stride), then plotting the mean height error per stride with respect to these two values as shown in Figure 9a,b. An observation is that the average height error is related to the level of heel strike, indicating that a higher heel strike impact leads to higher mean height error. This observation coincides with our hypothesis about heel strike error that bigger impact leads to more information loss, not only in velocity, but also in orientation estimation, and therefore larger error. It also helps explain the trial with large heel strike impact that needs slightly different thresholds for terrain determination.

The acceleration signal peak value and high-frequency acceleration energy percentage are also plotted as two groups of histograms in Figure 9c,d. The separation into two groups indicates that the heel strike signal on stairs is quite different than that on level ground, usually larger, meaning people land on the staircase harder than on level ground. This observation also demonstrates that it would not be appropriate to simply use the HS vertical velocity error on level ground to estimate that on stairs, when the fourth equation (known residual height) is not available to enable a unique solution for both acceleration bias and HS vertical velocity error.

### 7.4. Multiple Meanings of Acceleration Bias

As mentioned in Section 3, the acceleration bias in this model covers more than just accelerometer bias in the IMU error model. It also accounts for noise, error in sensor signal, and uncertainty in orientation estimation. The stable values of acceleration bias and HS velocity error on the level-ground walking demonstrate the existence of certain patterns in the parameters of the kinematic model. These parameters are likely a combination of true sensor errors and errors due to the repetitive gait motion. It is worth mentioning that the APDM sensor demonstrated good accuracy in static calibration, but still both the ESKF method and integration method generated non-zero acceleration bias. This is consistent with the assumption that the acceleration bias term is more than just the accelerometer bias. Other imperfections like bandwidth limitations and faulty detection of ZUPT can both contribute to the error. The multiple components lead to a wider, more blended distribution of acceleration bias in Figure 4 compared with HS vertical velocity error. 

### 7.5. Limitations

Although the proposed method showed good performance in our testing, some limitations remain. First, the proposed algorithm is a deterministic approach for reconstruction from IMU data that contains random noise; it may perform worse on sensors with higher noise levels. Although we do provide a parameter for handling different levels of sensor noise in the proposed algorithm, it may perform less effectively on sensors with increased noise, particularly if the noise is significantly greater than the bias term. Second, terrain determination relies on empirical selected fixed thresholds. It correctly classifies all terrain segments but is not sensitive enough to reliably detect the transition strides between level ground and ramps. Moreover, when applied on larger populations or on specific patient groups, patterns on those model outputs may vary and need adaptive thresholds. Third, the algorithm includes an assumption of zero height change that is appropriate for true level-ground walking, but it is violated for other terrains. The lack of information to estimate the error on non-level-ground walking led to using an assumed zero value of HS vertical velocity, which is not strictly correct. Other types of models for the proposed HS vertical velocity error were tested, including modeling the error as a Gaussian random variable or simply assigning the mean magnitude from other strides on level ground. However, these did not work well, for multiple potential reasons: the characteristic of heel strike event on ramps and stairs is different from that on level-ground walking as shown in Figure 9; thus, it does not make sense to approximate them with the data from level-ground walking; and moreover, HS vertical velocity error may simply be non-Gaussian. Additional consideration of how the different conditions of gait could generate valid assumptions to constrain the equations and generate measured, rather than assumed, values for HS vertical velocity error could lead to further improvement. Fourth, the algorithm as implemented assumes the HS vertical velocity error is an impulse in vertical velocity at a specific heel strike moment, while in reality it might be spread out over a short period of time. Alternative models for a distributed application of this impulse may also improve the reconstruction. Finally, the experiments used mostly a young and healthy test population, with only one case from a person with lower limb amputation using a prosthesis, in structured real-world indoor environments. Additional testing on populations with different characteristics will help verify that the method is generalizable and may further inform the selection of classification thresholds for general or population-specific use. There is also ongoing work to apply the proposed method to estimate real-world foot clearance in a clinical population with gait impairments—an application in which accuracy of the whole trajectory in the vertical direction is critical.

## 8. Future directions

One category of potential improvements is to handle the sensor noise better by modeling it probabilistically. For example, it may be possible to express the states’ uncertainty by a covariance matrix like a Kalman Filter, so that the algorithm could evaluate the contribution of the error model in the residual velocity and residual height adaptively, rather than with the fixed parameter C. Another possibility is to use the proposed error model as a plug-in for a Kalman Filter-based method to improve the performance of the Kalman Filter by providing estimation of HS vertical velocity error and acceleration bias. In either case, it may be appropriate to correct different amounts in the x, y, and z directions by using separate values of C or Kalman gains. Additionally, it may be worthwhile to study the distribution of acceleration bias and HS vertical velocity error and model the kinematics using statistically derived values for the unknown parameters on ramps and stairs. 

**Figure 10:**
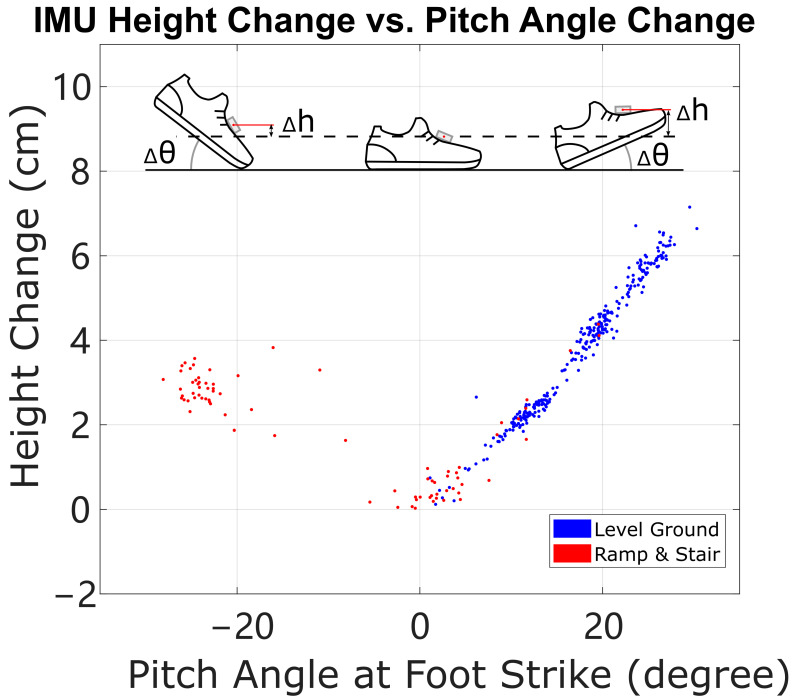
The change of height from foot strike to foot-flat phase vs. pitch angle change from foot strike to foot-flat. The angular motion of the foot and the linear motion of the IMU should be related through the kinematics of the foot lever. Both positive values of pitch angle (typical heel strike) and negative values (forefoot strike, mainly from down-stairs strides) show evidence of the expected linear scaling.

Another category of possible improvements is to find additional constraint equations to allow measurement-derived HS vertical velocity errors on non-level terrain, instead of an assumed zero value or a statistical value. As discussed in Section 5, the lack of additional information forbids a unique solution for the error states on these terrains. However, it may be possible to use the kinematics of the foot and shoe to generate another kinematic constraint, such as a relationship between linear and angular motion between heel strike and foot-flat, or between foot-flat and toe-off. For example, suppose the geometry of the shoe can be obtained with a 3D scanner, and the deformation of the shoe can be handled properly; then the motion of the foot-mounted IMU can be estimated using shoe geometry and angular motion as long as it is in contact with the ground during heel strike phases [18]. If the IMU’s height can be recorded just before heel strike, before it has been polluted by heel strike error, and then recorded again as a final height during stance phase, then this motion of the shoe “tipping down” from foot strike to foot-flat could create another kinematic constraint equation, and we can compare the computed height change of the IMU with its estimated motion from shoe geometry and angular motion. Figure 10 shows an example implementation of this relationship: a graph of the change in IMU height with respect to changes in pitch angle from foot strike to foot-flat. We use “foot strike” here because when walking on stairs, subjects may either strike the staircase with heel or forefoot. The slope on the right line should be the posterior distance between the end of the shoe to the position of the IMU. The left line is a similar computation around the time of forefoot strike, e.g., for some stair strides; the slope of the line should be the anterior distance from the tip of the shoe to the position of the IMU. These and other sources of constraint equations related to foot motion could be topics for further exploration. 

In addition, the new method could be expanded to incorporate additional information about known terrain or terrain consistency. The ramps and stairs reconstruction here reverted to a simple elimination of acceleration bias because it assumed no knowledge of the ground. However, past work has shown utility in assuming specific terrain characteristics such as the height of each stair [2], if known. Because the final height of the footfall is precisely the information needed to perform the HS vertical velocity error estimation, the same information could be incorporated into the proposed method. Ramps of known slope could be handled similarly, with final height determined by known slope and calculated stride length, perhaps by separating horizontal and vertical correction steps in the algorithm. It may also be possible to relax the assumption of known terrain and instead assume only internal consistency. Most ramps and stairs in the built environment have constant slope or stair height, so it may be feasible to separate them into different bouts or locations and back-correct distinct sets of strides to satisfy an assumption of constant slope or fixed increments of stair height, even if the value of this slope or stair height is not known a priori.

Finally, the proposed method in general assumes strides to be independent except when verifying the consecutive ramps/stairs condition in terrain determination. This feature makes it easy to speed up data processing using parallel computing techniques. Unlike the Kalman Filter-based method, which is naturally a sequential algorithm, our method breaks down the data dependency into a stride-by-stride level. For post-processing type of task, for example, gait analysis after in-field data collection, all strides can be reconstructed with CPU-based parallel computing for Algorithms 1 and 2 before entering the next stage in the pipeline. This capability of speeding up is of great importance as data are becoming more available than ever before. On the other hand, in some circumstances it might be beneficial to incorporate temporal connection of the model outputs like the state machine in [9] that adopt adaptive thresholds based on the current and next terrain type, after quantifying the model outputs with more thorough understanding.

## 9. Conclusions

To improve the reconstruction accuracy in the height direction and determine terrain types using only one foot-mounted IMU, this work proposed a kinematic model that estimates a constant acceleration bias and impulsive heel strike vertical velocity error by assuming zero residual velocity and zero height change as if on level ground. By examining the violation of the zero height change assumption, the proposed method can determine whether an individual stride occurred on level ground, ramp, or stair terrain by simply thresholding the output HS vertical velocity error. Compared with ESKF method and ZUPT method, the proposed method can explicitly estimate the error caused by heel strike impact on level-ground walking. The new approach achieves significant improvement in height drift on level ground and moderate improvement on ramps and stairs as well. 

## Figures and Tables

**Table 1:** Confusion matrix of terrain determination results for 15 trials.

Terrain Types		Predicted		
		Level Ground	Ramps	Stairs
Ground Truth	Level ground	4290	1	0
	Ramps	18 ^1^	432	0
	Stairs	0	0	1268

^1^ Seventeen of eighteen strides are transition strides on the edge of ramps.

**Figure A1:**
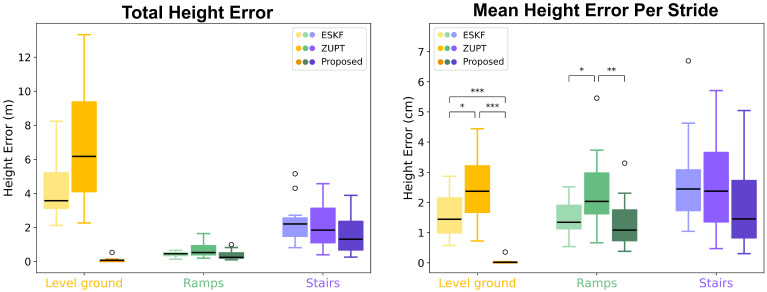
Total height error and mean height error per strides from reconstruction without Kalman Filter-based orientation estimation (*t*-test values: * *p* < 0.05; ** *p* < 0.01; *** *p* < 0.001).

## Data Availability

Dataset available on request from the authors.

## Appendix A. Reconstruction Results with Orientation Computed Using a Non-KF Method

The IMU orientation information used in the proposed method can be obtained from any proper orientation estimation algorithm. In this appendix, the reconstruction results using a non-Kalman Filter-based orientation method are presented, and the height error on three terrains is compared using the proposed reconstruction method compared against the ESKF method and ZUPT method.

The orientation estimation has two components: quaternion-based strap-down integration [11], and inclination correction using accelerometer data [3,10,40,62]. The strap-down integration simply follows integration equations derived from the quaternion kinematics, as shown in Equation (5). When the IMU is considered under small motion, we can assume that the accelerometer measures only gravity; thus, the accelerometer reading can be used to correct the quaternion by computing the misalignment of the inclination angle. In practice, we add a small gain to the inclination correction to increase robustness, similar to method 3 in [40].

The mean value and the statistical significance of the differences in mean height error per stride among the three methods based on two-tail two-sample Student’s t-tests are shown in Figure A1. The height error in both integration-based methods increases, due to the reduced accuracy of the non-Kalman Filter-based orientation estimation method compared with the Kalman Filter-based method. However, the proposed method still performs the best among three methods, demonstrating robustness to the orientation estimation results. In contrast, the height error of the ZUPT method increased significantly, and became the worst among the three methods. Because the ESKF version includes orientation estimation that cannot readily be separated out, nothing changed in the ESKF method here and the height error of ESKF remained the same as in Figure 6.
